# Medicinal Plants and Plant-Based Remedies in Grande-Terre: An Ethnopharmacological Approach

**DOI:** 10.3390/plants12030654

**Published:** 2023-02-02

**Authors:** Elisa Courric, David Brinvilier, Petra Couderc, Alejandro Ponce-Mora, Vanessa Méril-Mamert, Muriel Sylvestre, Jeannie Hélène Pelage, Jean Vaillant, Alain Rousteau, Eloy Bejarano, Gerardo Cebrian-Torrejon

**Affiliations:** 1 COVACHIM-M2E Laboratory EA 3592, Department of Chemistry, University of the French West Indies, Fouillole Campus, UFR SEN, CEDEX, 97157 Pointe-à-Pitre, France; 2 Department of Biomedical Sciences, School of Health Sciences and Veterinary, Universidad Cardenal Herrera-CEU, CEU Universities, 46113 Moncada, Spain; 3 Départament de Medicine Générale, Faculté Hyacinthe Bastaraud, University of the French West Indies, Fouillole Campus, 97157 Pointe-à-Pitre, France; 4 LAMIA, EA 4540, Department of Chemistry, University of the French West Indies, Fouillole Campus, UFR SEN, CEDEX, 97157 Pointe-à-Pitre, France; 5 UA, UMR EcoFoG, CNRS, Cirad, INRA, Université des Antilles, Université de Guyane, Université des Antilles, 97159 Pointe-à-Pitre, France

**Keywords:** ethnobotany, ethnopharmacology, biology of medicinal plants

## Abstract

The island of Grande-Terre is a French overseas region that belongs to the Guadeloupean archipelago, a biodiversity hotspot with unique flora. Herbal medicine is widely used in the island for therapeutical purposes; however, there is a significant knowledge gap in the records relating to medicinal plants and their associated uses. Ethnobotanical survey methodology using quantitative parameters (informant consensus factor, species use value, relative frequency of citation, frequency use of a treatment and plant for an ailment) provided insights into the traditional medicinal use of a given plant. Ninety-six different plant species distributed among 56 families were identified and 523 remedies were documented in the survey. After data filtering, 22 plants species were associated with 182 remedies. The most frequent plant families were Poaceae, Myrtaceae, Cucurbitaceae and Rubiaceae. Aerial parts of these plants were the most common parts of the plant used for the remedies and the most frequent mode of administration was oral ingestion. This study highlights a valuable traditional knowledge of folklore medicine and helps to document and preserve the association of a plant with—and its use frequency for—a given ailment. These findings might be the starting point for the identification of biologically active phytocompounds to fight common health debilities.

## 1. Introduction

According to the World Health Organization (WHO), traditional medicine represents the only way to access medical care for millions of people the world over [1]. In this context, herbal medicine arises as one of the most used forms of complementary and alternative medicine [2]. Herbal medicine, also known as phytotherapy, refers to the use of plants or plant derivates to prevent or treat a given disease. In many cultures, herbal medicine has been traditionally used to treat a wide range of ailments such as cardiovascular affections, cancer, neurodegenerative disorders, and diabetes mellitus [3,4,5,6,7].

Ethnobotanical studies enable the value of traditional knowledge to be better appreciated, by raising the awareness of the local population about the importance of preserving their know-how and by strengthening the link between the population and its environment. In recent years, the interest in ethnopharmacology and pharmacognosy has increased considerably, and numerous ethnobotanical studies have been conducted throughout the world [8,9,10,11]. Interestingly, the number of countries with regulations on herbal medicines has been increasing over the last few years. In fact, the number of member states of the WHO with national or state-level laws regarding traditional and complementary medicine increased from 45 to 109 between 1999 and 2018 [12]. In addition, ethnopharmacological analysis is one of the first steps in the development of new pharmaceuticals and the identification of new active molecules [13,14,15,16]. In the Caribbean island of Guadeloupe, a biodiversity hotspot, herbal medicine is traditionally used to cure ailments. However, the documentation of Guadeloupean folklore medicine is scarce. In this study, an ethnobotanical survey was conducted to provide a snapshot of the herbal folk medicine knowledge on the Grande-Terre Island in Guadeloupe.

Plant-based drugs and traditional treatments are often more accessible, less onerous, and more affordable than conventional drugs. It is estimated that around 80% of the population of developing and low-income countries uses therapeutic plants for basic healthcare [17]. Interestingly, many patients choose to combine natural products and herbal medicine with their pharmaceutical medication to mitigate their possible side effects [18,19].

Guadeloupe, an archipelago consisting of six groups of islands in the Lesser Antilles at the junction of the Caribbean Sea and the Atlantic Ocean, represents an important reservoir of floral biodiversity. Guadeloupe has unique botanical peculiarities: 1709 species of native vascular plants of which twenty-one species are endemic to Guadeloupe, more than 20% of the Guadeloupean tree species are endemic to the Lesser Antilles, and more than 250 species of vascular plants in Guadeloupe are threatened [20,21]. Due to these botanical singularities, Guadeloupe is included in the Caribbean Islands biodiversity hotspot, one of the thirty-five biodiversity hotspots in the world [22].

**Figure 1:**
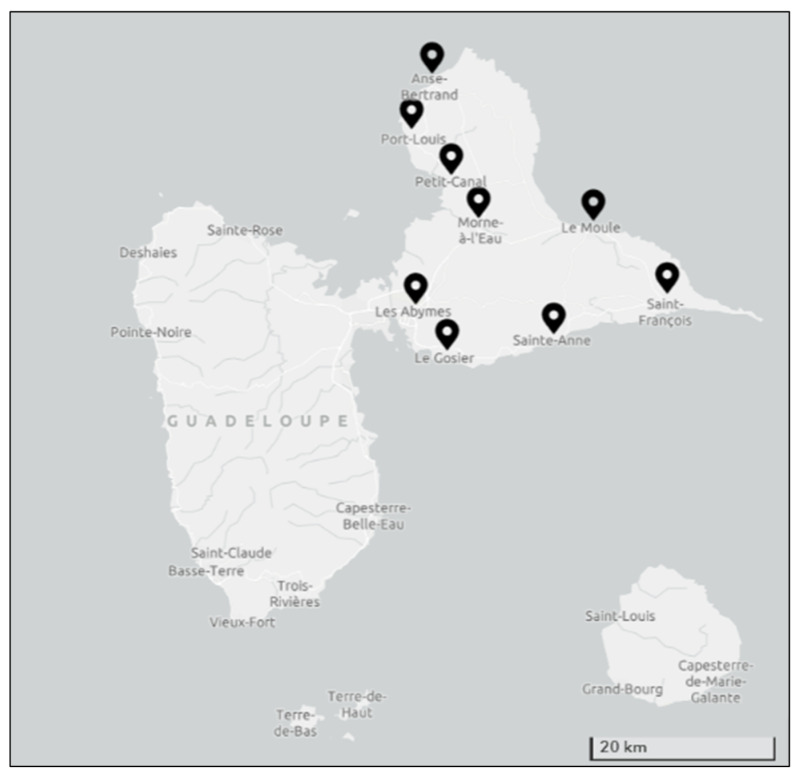
Geographical position of Grande-Terre. Nine different municipalities of Grande-Terre Island where the survey was conducted are shown in the map.

The two main islands, known as Basse-Terre and Grande-Terre, are separated by a narrow sea channel (Figure 1). Basse-Terre is the largest island in the archipelago and is of volcanic origin. The island contains a north-south oriented mountain range whose ridge line reaches 1000 m. Due to its location, Basse-Terre is sheltered from leeward winds, and it is partially covered by rainforests. The island of Grande-Terre, where the present survey was carried out, is mainly flat and is constituted of eroded and fragmented limestone plateaus. On this land, the vegetation experiences a dry season of several months. The climate of Grande-Terre is considered tropical maritime with an annual temperature between 20 °C and 32 °C and annual rainfall between 1500 and 2000 mm. In the karst valleys, there remain relics of seasonal evergreen forests. On the western coast, the island comprises swamp forests and mangroves. At present, a secondary dry forest covers only 22% of the territory, but the original vegetation is being partially removed by agricultural activities and deforestation. Grande-Terre is the island with the highest density of population of the Guadeloupean archipelago with 224,901 inhabitants reported on 1 January 2016.

In this geographic area, a large proportion of the population has limited access to healthcare, and the primary care system is often burdened due to the high prevalence of infectious and parasitic diseases. Patients’ treatment often relies on popular remedies and herbal medicine, at least as an initial therapeutic step. This helps to explain the current relevance of traditional medicine in the archipelago despite the progress of modern medicine. Guadeloupe cherishes particularly diverse plant-based remedies, as a heritage from the European, African, and Indian cultures [23]. Unfortunately, due to lifestyle modification, oral transmission from one generation to another may be disrupted, and thus, traditional knowledge might be weakened. Ethnobotany and ethnopharmacology represent two useful tools for the preservation of herbal medicine knowledge and the conservation of cultural patrimony. Of note, ethnobotanical surveys represent the most efficient mechanism through which to preserve traditional medicine as well as being the starting point for the identification and characterization of bioactive phytocompounds for therapeutic use.

TRAMIL (Program of Applied Research to Popular Medicine in the Caribbean) is a network program of applied research on the traditional and medicinal plant resources of the Caribbean Basin region. This program has established the guidelines in Caribbean ethnobotanics and has inspired multiple research projects [23,24,25,26,27]. However, to date, only limited information is available about the medicinal uses of plants by the local population in Guadeloupe [23].

In this study, we explored folklore medicine in Grande-Terre, one of the two main islands of Guadeloupe. An ethnobotanical survey by face-to-face interviews was conducted following the TRAMIL-inspired methodology, and a quantitative analysis allowed us to identify plant species and methods of preparation used to cure ailments in this geographic region. Here we report eight ailments and 22 different plant species that adequately outline the current knowledge in herbal medicine in Grande-Terre.

## 2. Results

### 2.1. Profiles of Medicinal Plant Users in Grande-Terre

A total of 118 participants in nine different regions of the island were interviewed in the study, which respectively correspond to a representative fraction of the population and 0.05% of the survey area. Note that the minimum number of participants (n) to interview was estimated as 44 [28], that is, only 0.02% of the population of Grande-Terre. The average age of the participants was 53 years old, ranging from 18 to 99 years old. Of the participants, 66.9% were women (89/118) and 33.1% were men (39/118). Regarding the living area, 78.8% of the participants came from the countryside (93/118), and 22.2% (25/118) were from the city. Regarding the ease to carry out the interview, no differences were noticed between rural and urban areas. Most participants were of African origin (72.9% 86/118); 10.2% (12/118) were of mixed African and European origins; 5.9% (7/118) were of mixed African and Indian origins; (5.1%) 6/118 were of Indian origin; 3.4% (4/118) were of European only origin; 1.7% (2/118) were of mixed African, European, and Indian origin; and only 0.8% (1/118) were of Haitian origin.

Participants were asked about the source of their knowledge regarding herbal remedies. Only 3.4% (4/118) of the participants declined to answer this question. Information given by participants on the uses of plants with medicinal properties was predominantly sourced from family (56.8% or 67/118). Some participants (2.5% or 3/119) acquired their knowledge from family and acquaintances, others from acquaintances only (5.9% or 7/118); a greater number acquired their knowledge from a mix of family and personal research (19.5% or 23/118) than from their personal research only (3.4% or 4/118). Knowledge about plants came also from elders (5.9% or 7/118), being gleaned in the market (1.7% or 2/118), or from dreams and God (0.8% or 1/118). In the family category, the cited sources were grandparents (32.5% or 41/126), parents (26.2% or 33/118), mother (13.5% or 17/126), grandmother (9.5% or 12/118), step-parent (3.2% or 4/118), stepmother (2.4% or 3/118) and unspecified relatives (11.9% or 15/118). In the personal research category, the sources of information cited were books (35.3% or 12/34), the Internet (26.5% or 9/34), the radio (17.6% or 6/34), specialists (11.4% or 4/34) and television (8.8% or 3/34). Regarding the origin of the medicinal plant, 68.6% of the participants (81/118) collected the plants from their garden, 3.4% (4/118) from a combination of their garden and the market, 3.4% (4/118) only from the market, 2.5% (3/118) from a combination of their own garden and the near neighborhood areas, and 6.8% (8/118) away from home. For 18 participants (15.3%), the plant origin was unknown.

### 2.2. Identification of Plant Species and Herbal Remedies in Grande-Terre

**Figure 2:**
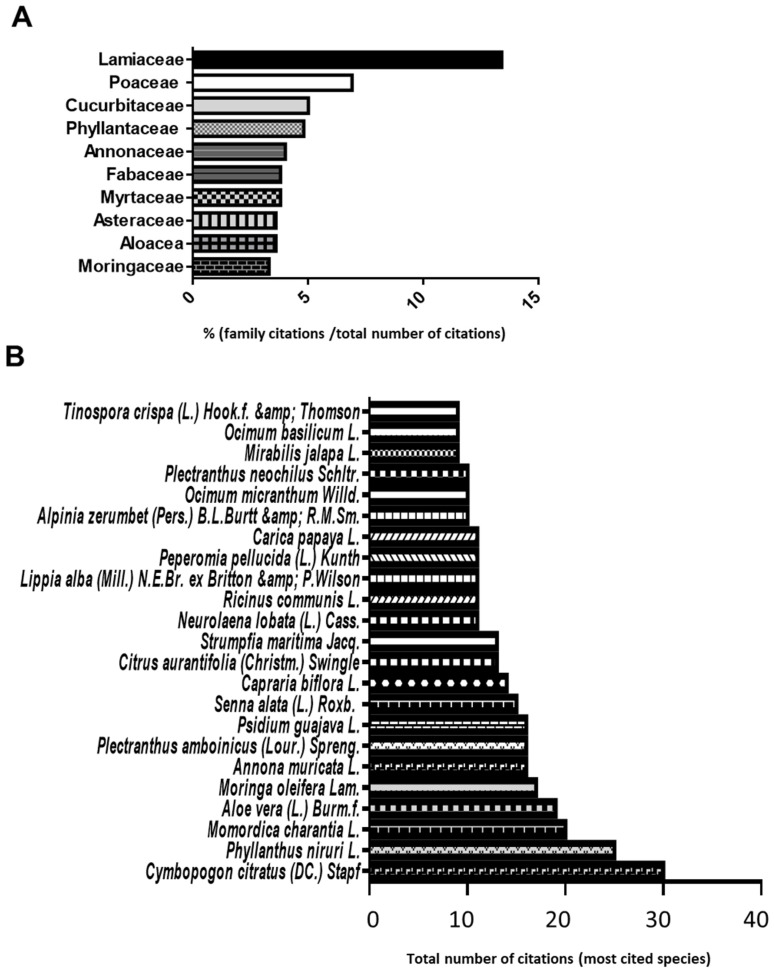
Medicinal plants identified in the ethnopharmacological survey: (**A**) botanical families predominantly used in Grand-Terre and (**B**) number of citations for each plant.

Ninety-six different plant species distributed in 56 families were identified and a total of 523 remedies were documented. The most frequent plant families were Lamiaceae (13.4% or 70/523), Poaceae (6.9% or 36/523), Cucurbitaceae (5% or 26/523), Phyllantaceae (4.8% or 25/523), Annonaceae (4% or 21/523), Fabaceae (3.8% or 20/513), Myrtaceae (3.8% or 20/523), Asteraceae (3.6% or 19/523), Aloacea (3.6% or 19/523) and Moringaceae (3.3% or 17/523) (Figure 2A).

Respectively: *Plectranthus amboinicus* (Lour.) Spreng. (22.9% or 16/70) was the most cited of Lamiaceae; *Cymbopogon citratus* (DC.) Stapf (83.3% or 30/36) of Poaceae; *Momordica charantia* L. (76.9% or 20/26) of Cucurbitaceae; *Annona muricata* L. (76.2% or 16/21) of Annonaceae; *Senna alata* (L.) Roxb. (75% or 15/20) of Fabaceae; *Psidium guajava* L. (80% or 16/20) of Myrtaceae; and *Neurolaena lobata* (L.) Cass. (57.9% or 11/19) of Asteraceae. *Phyllanthus niruri* L. (100% or 25/25) was the only cited species of Phyllanthaceae, *Aloe vera* (L.) Burm.f. (100% or 19/19) was that of Asphodelaceae, and *Moringa oleifera* Lam. (100% or 17/17) that of Moringaceae (Figure 2B).

**Figure 3:**
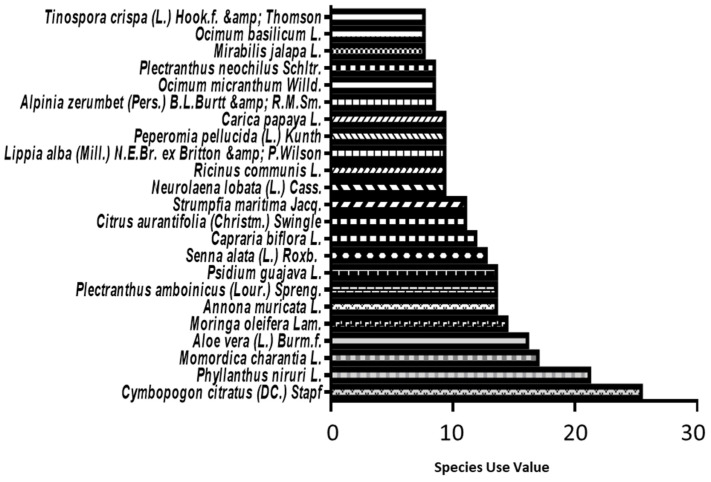
Medicinal use value of plant species commonly used in traditional folk medicine in Grand-Terre.

To evaluate the relative relevance of a plant species in folklore medicine in the area of study, we used the use value (UV) [29,30]. *Cymbopogon citratus* (DC.) Stapf (5.7% or 30/523), *Phyllanthus niruri* L. (4.8% or 25/523), and *Momordica charantia* L. (3.8% or 20/523) were the most cited plants and had the highest UV (Figure 3). *Cymbopogon citratus* (DC.) Stapf is used by more than 1 out of 4 participants (UV = 25.4% or 30/118), *Phyllanthus niruri* L. is used by more than 1 participant out of 5 (UV = 21.2% or 25/118) and *Momordica charantia* L. is used by more than 1 out of 6 participants (UV = 16.95% or 20/118).

### 2.3. Plant-Based Remedies in Grande-Terre

**Figure 4:**
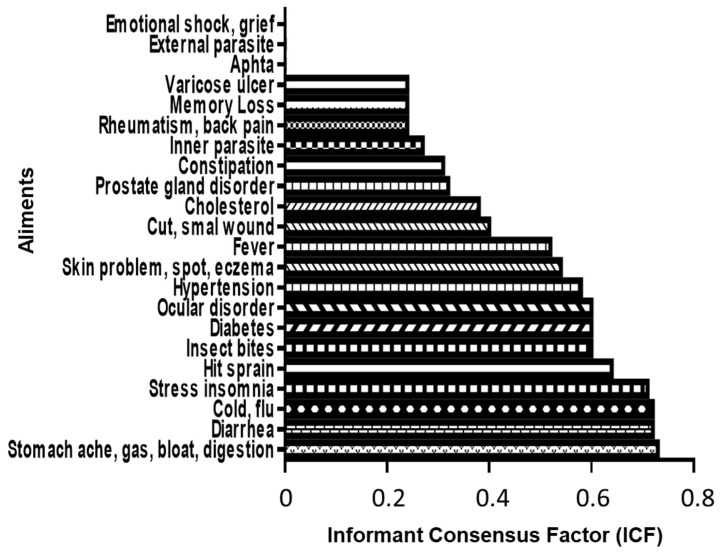
Alignment of categories in the ethnopharmacological survey. Informant consensus factor (ICF) is shown for each alignment.

A total of 523 remedies were documented in our survey and the informant consensus factor (ICF) index was used to evaluate the homogeneity of the participants’ knowledge [31]. The highest ICF of 0.73 was scored for “Diarrhea” followed by “Digestive problems, bloating and stomach ache” and “Cold, flu” with an ICF of 0.72; “Stress, insomnia” scored an ICF of 0.71, while “Injury, sprain” the ICF value was 0.64 (Figure 4). The lowest ICF was reported for “Emotional shock, grief”, “Aphtha” and “External parasite” with a value of 0. The original data was filtered using two quantitative ethnobotanical parameters to prioritize the most relevant information. First, to assure a minimal homogeneity in the participants’ knowledge, only ailments with an ICF > 0.55 were selected. Secondly, the data were filtered using the frequency Fa which indicates if an ailment is commonly cured with plants, and also frequency Fi which determines the most frequently used plant species to treat a particular ailment [32,33]. To avoid the analysis of anecdotal plants, we extracted from the list only species with a Fi ≥ 8%, and only ailments with a Fa > 3% were kept, supporting a common use of these plants to cure the ailments. Only 8 out 22 ailments fulfill the selection criteria: cold/flu; hit/sprain; stomach ache/gas/bloating/digestion; diarrhea; insect bites; diabetes; stress/insomnia and hypertension. Only 22 species belonging to 20 families and only 182 remedies remained in the final selection after filtering (Table 1). Among these 22 plants cited as remedies, the more frequent families were: Lamiaceae (14.8% or 27/182), Poaceae (13% or 24/182), Myrtaceae (10.4% or 19/182), Cucurbitaceae (8.2% or 15/182) and Rubiaceae (6.6% or 12/182).

**Figure 5:**
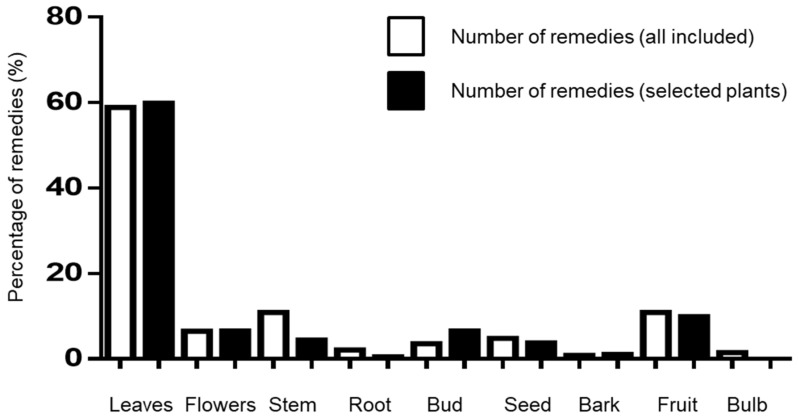
Plant-based remedies in the ethnopharmacological survey. Percentage of remedies using different plant parts for 523 initials remedies involving only the 22 plants selected.

The plant preparations used as herbal remedies comprise different plant parts, including leaves, flowers, fruits, stems, buds, roots, bark, seed, or bulbs (Figure 5). For the eight ailments and 22 plants selected, aerial parts are used in 85.7% of the cases: leaves in 59.9% of the remedies (109/182), stems mixed with flowers and leaves in 7.1% of the remedies (13/182), both flowers alone and buds alone in 6.6% of the remedies (12/182), stems in 4.4% of the remedies (8/182), and bark only in 1.1% of the herbal preparations (2/182). Reproductive parts are used in 13.7% of the remedies: fruits in 9.9% (18/182), and seeds in 3.8% (7/182). The underground plant parts are used in 0.6% of the remedies, of which solely the roots are used.

**Figure 6:**
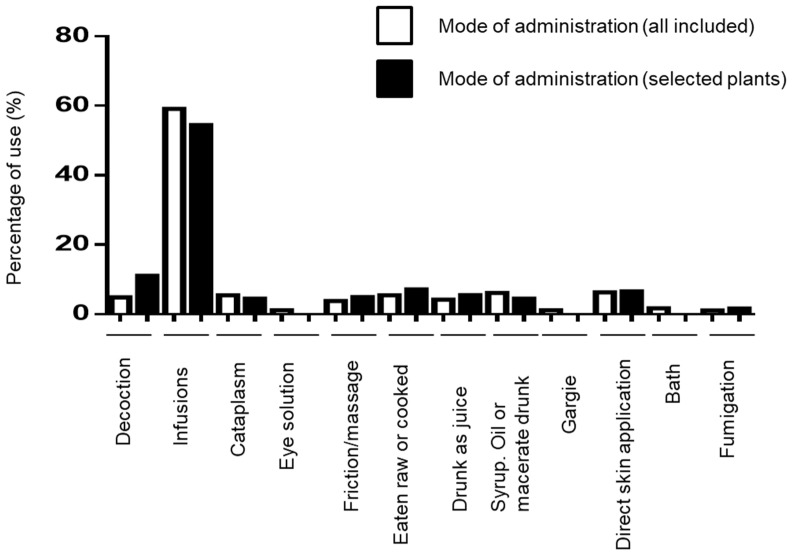
Mode of administration in Grande-Terre. Percentage of different mode of administration for 22 selected plants and all remedies included.

Several ways to prepare plants were mentioned including infusions, juices, maceration-derived preparations, decoction-derived preparations, syrups, and oils—and the plants might be raw, mashed, or cooked (Figure 6 and Table 2). Once prepared, remedies are applied in multiple forms: (I) decoctions, infusions, juices, syrups, macerates, or edible plants are ingested orally; (II) juices, macerates, or poultices can be applied directly to the skin by friction, massage, or direct contact; (III) eye solution preparations; and (IV) plants can be used to fumigate a specific area as prevention for insect bites. Infusions are prepared by soaking plant material in hot water for between 10 min and several hours. For decoctions, the participants boil the plant in water until the volume of liquid is reduced to more than 1/2 or 3/4 of the original amount of liquid. Macerations are obtained by soaking the plant material in cold water for a few hours or in alcohol for several days. Syrups are prepared by putting the plant in sugar for one night. The plants prepared in raw form are used immediately after harvesting. For the eight ailments and 22 plants selected, the oral route is the most frequent mode of administration in 82.4% of the total remedies: infusions are used in 54.4% of the total remedies (99/182); decoctions in 11% of the cases (20/182); raw or cooked plants in 7.1% (13/182); juices in 5.5% (10/182); and syrups, oils or macerated preparations in cold water or alcohol in 4.4% (8/182). Skin application is used in 15.9% of the remedies: direct application on the wound in 6.6% (12/182), friction or massage in 4.9% (9/182), and poultice in 4.4% (8/182). Fumigation is used in 1.6% (3/182) of the remedies (Figure 6).

#### 2.3.1. Digestive Problems, Bloating, and Stomach Ache

Thirty different plants were mentioned in 107 remedies to cure stomach aches, gas, bloating, or to help with digestion. The three most cited plants were *Strumpfia maritima* Jacq. (11.2% or 12/107), *Capraria biflora* L. (9.3% or 10/107) and *Momordica charantia* L. (8.4% or 9/107) (Table 1). To help digestion, and reduce intoxication or a stomach ache, *Strumpfia maritima* Jacq., also called “womarenbòlanmè” is used. An infusion is prepared with the leaves of this indigenous plant and one cup of the preparation must be taken after the meal or at night. Leaves can also be marinated in rum and, in the case of serious pain, a spoonful of the preparation is taken (Table 2). *Caprari abiflora* L., known as “tépéyi”, is an indigenous plant with properties against liver disorder, digestion problems, or stomach aches. A cup of infusion is prepared using three leaves or small stem parts. The infusion can be taken in the morning, at night, or several times during the day (Table 2). Leaves of *Momordica charantia* L., a plant also referred to as “pawoka”, are also used to prepare infusions. One cup is taken once or twice a day in case of digestive disorders, liver problems, and hangovers (Table 2).

#### 2.3.2. Diarrhea

Eight different plants were mentioned in 27 remedies to cure diarrhea. The main plant used is *Psidium guajava* L. (59.3% or 16/27), also called “goyavier” (Table 2). Two or three young leaves or buds are necessary to make one cup of infusion. The dose depends on the severity of the symptoms, although participants recommend no more than two or three cups a day to avoid the risk of constipation (Table 2).

#### 2.3.3. Cold and Flu

Ninety-five plant remedies and twenty-seven different plant species were mentioned to cure colds and flu. The three plants most commonly used, with a Fi > 8%, are *Cymbopogon citratus* (DC.) Stapf (20% or 19/95), *Plectranthus amboinicus* (Lour.) Spreng. (11.6% or 11/95) and *Alpinia zerumbet* (Pers.) B.L.Burtt & R.M.Sm. (8.4% or 8/95) (Table 1). From *Cymbopogon citratus* (DC.) Stapf, commonly referred to as “ti can”, “citronelle” or “sitwonnèl”, the leaves are used to prepare an infusion, and one cup must be taken at night before going to bed (Table 2). The leaves of *Plectranthus amboinicus* (Lour.) Spreng., called “gwo ten”, are also used to prepare an herbal remedy. Two or three leaves are employed to make one cup of infusion. It must be taken one or two times a day, at night or during the day, for 1 or 2 days (Table 2). To cure the flu, leaves, flowers, or aerial parts of *Alpinia zerumbet* (Pers.) B.L.Burtt & R.M.Sm., called “atoumo”, can be used. They are also prepared in infusion and a cup must be taken two or three times a day (Table 2).

#### 2.3.4. Stress, Insomnia

Six different plants were mentioned in 18 remedies to cure stress and insomnia. The three plants mainly cited were *Annona muricata* L. (61.1% or 11/18), *Lippia alba* (Mill.) N.E.Br. ex Britton & P.Wilson (11.1% or 2/18), and *Cymbopogon citratus* (DC.) Stapf (11.1% or 2/18) (Table 1). The leaves of *Annona muricata* L., also known as “howosòl”, are used to prepare an infusion that must be taken at night before going to sleep. Leaves of *Lippia alba* (Mill.) N.E.Br. ex Britton & P.Wilson and *Cymbopogon citratus* (DC.) Stapf are also used (Table 2).

#### 2.3.5. Hit, Sprain

Twenty-nine herbal remedies, using 11 different plants, were cited to treat muscular aches, hits or sprains. The three plants most commonly cited were *Mirabilis jalapa* L. (65.5% or 9/29), *Carica papaya* L. (24.1% or 7/29), and *Justicia pectoralis* (13.8% or 4/29) (Table 1). *Mirabilis jalapa* L, known as “belle de nuit”, is always used as a poultice to relieve sprains. Leaves are mashed, mixed with salt, and sometimes with rum or vinegar. The mixture is directly applied to the skin, maintained with a bandage, and changed every day until the injury is healed (Table 2). *Carica papaya* L., called “papayer” or “pyépapay”, is used to relieve injuries or muscular aches. The male flowers are macerated for several days with bay rum, a distillate made with rum and leaves or berries of *Pimenta racemosa*. The preparation is applied directly on the skin and accompanied by a massage or friction. Leaves of *Justicia pectoralis*, an indigenous plant called “zèbchapantyé”, are used in the same way to cure sprain. The leaves are applied as a poultice or with castor oil or salt (Table 2).

#### 2.3.6. Insect Bites

To reduce or prevent insect bites, 16 different plant remedies comprising seven plant species were mentioned. The four plants mainly cited were *Aloe vera* (L.) Burm.f. (25% or 4/16), *Citrus aurantifolia* (Christm.) Swingle (25% or 4/16), *Cymbopogon citratus* (DC.) Stapf (18.8% or 3/16) and *Pimenta racemosa* (12.5% or 2/16) (Table 1). Participants state that the gel contained in *Aloe vera* (L.) Burm.f. leaves is directly applied on insect bites to alleviate itch or pain. *Citrus aurantifolia* (Christm.) Swingle juice is applied directly to the bite. Leaves of *Cymbopogon citratus* (DC.) Stapf have a preventive role and are used in fumigation to repel insects. Finally, preparations of *Pimenta racemosa* can be directly applied to the skin. The preparation of this plant species, whose vernacular name is “boisd’inde”, is made of leaves and berries that are macerated in rum.

#### 2.3.7. Diabetes

For the treatment of diabetes, 48 plant-based remedies and 20 plant species were documented. The four most cited plants were *Moringa oleifera* Lam. (18.8% or 9/48), *Tinospora crispa* (L.) Hook.f. & Thomson (16.7% or 8/48), *Phyllanthus niruri* L. (12.5% or 6/48) and *Momordica charantia* L. (12.5% or 6/48) (Table 1). *Moringa oleifera* Lam. seeds are usually eaten in the morning to manage diabetes. Furthermore, the plant leaves can be cooked and eaten as vegetables and can also be used to make an infusion that can be drunk every morning on an empty stomach (Table 2). Small parts of *Tinospora crispa* (L.) Hook.f. & Thomson, also known as “lyannsépan”, can be infused with water to make a remedy against diabetes. The infusion can be taken once a day, in the morning, or all day long for several days (Table 2). Leaves of *Phyllanthus niruri* L., also knowns as “grenn an ba fey”, are used to prepare an infusion for drinking. Two or three leaves are used to make one cup of infusion, to drink periodically. The participants highlighted that this herbal remedy can also be used in case of acute crisis, but no more than two days in a row. Finally, the leaves and fruits of *Momordica charantia* L. are also used to manage diabetes; its leaves are also sometimes cooked as a vegetable and eaten with rice. A handful of leaves or one fruit of the plant is used to prepare one cup of infusion, to drink once a day (Table 2).

#### 2.3.8. Hypertension

Thirty-eight plant remedies comprising 16 different plant species were documented to reduce high blood pressure. The four most mentioned plants were *Passiflora edulis* f. flavicarpa (13.2% or 5/38), *Spondias cytherea* Sonn. (13.2% or 5/38), *Peperomia pellucida* (L.) Kunth (10.5% or 4/38) and *Terminalia catappa* L. (10.5% or 4/38) (Table 1). To treat hypertension, fruits of *Passiflora edulis* f. flavicarpa can be eaten raw or consumed in juice once a day. Another reported way of using the plant is to macerate half a fruit in warm water and drink one cup a day for three days. Fruits of *Spondias cytherea* Sonn., a plant also known as “pòmnsitè”, are consumed in juice from time to time during the day. Leaves of *Peperomia pellucida* (L.) Kunth, known as “koklaya”, are consumed raw, in salads, or prepared in infusion. Two or three are used to make one cup, to be drunk during the day (Table 2). Finally, *Terminalia catappa* L. is commonly found in streets or gardens in Guadeloupe and seems to be related to hypertension treatment. People suffering from hypertension put one red or yellow leaf in their socks, under the arch of the foot, or on their head under their cap, all day long. Leaves are also used in decoctions: half of a leaf is needed to prepare one cup of a preparation that must be drunk once a day (Table 2).

## 3. Discussion

The current ethnobotanical research covers a significant knowledge gap in the record of traditional therapeutical uses of plants in Grande-Terre. The ethnobotanical survey methodology and quantitative parameters used here result in an informative study on medicinal plants and plant-based remedies used in herbal medicine in the Guadeloupean archipelago, a biodiversity hotspot with a unique flora. Our research highlights a valuable traditional knowledge of folklore medicine and helps to document and preserve the association between a plant and a given ailment, and the frequency of its use in treatment.

Ninety-six different plant species were distributed across 56 families and 523 different remedies. Lamiaceae, Poaceae, Cucurbitaceae, Phyllantaceae, Annonaceae, Fabaceae, Myrtaceae, Asteraceae, Aloacea, and Moringaceae were the most prominent plant families for herbal remedy preparation among the population of Grande-Terre. These families include several perennial plant species that are commonly cultivated, found in the wild, or present in family gardens in Guadeloupe. These results are consistent with the findings of other ethnobotanical studies performed in the Caribbean basin. In Trinidad, the major plant families used in herbal medicine were Asteraceae, Lamiaceae, Leguminosae, Verbenaceae, and Poaceae [53]; in another study carried out in Jamaica, Fabaceae, Lamiaceae and Asteraceae were the most cited plant families regarding herbal folk medicine [24]. In a Puerto Rican study, Laminacea, Verbenaceaea, Asteraceae, and Annonaceae are also among the most frequent plant families used in plant-based medicine [26]. Moreover, a previous ethnobotanical study in Les Saintes points out that Fabaceae and Lamiaceae were the two plant families most used in herbal remedy usage [23]. These findings suggest a certain homogeneity regarding the usage of plant families within the Caribbean basin. However, depending on the ethnic background of the Caribbean population, plant use may differ [25]. The findings of the current study have some similarities but also differences with comparable ethnobotanical investigations carried out in other parts of the world with different geobotanical properties. This is not surprising because the availability of the different species is dependent on climate and soil conditions, and plants can be chosen on the basis of taste, beauty, scent and so on. Thus, for example, Laminaceae, Asteraceae, and Rosaceae seem to be strongly correlated to herbal preparations in the Mediterranean area [54], while Poaceae, Asteraceae, Fabaceae, Liliaceae, and Ranunculaceae are the main plant families used in Chinese traditional medicine [55].

*Cymbopogon citratus* (DC.) Stapf was the most mentioned plant species in our ethnobotanical survey. Known as Indian verbena, the plant is native to India, where it is used as a medicinal plant [56]. Its use in Guadalupe represents a legacy of Indian culture and demonstrates how migration-related events can result in the transmission of traditional knowledge from one society to another. The present study indicates that, in Grande-Terre, the local population uses *Cymbopogon citratus* to treat cold, flu, insect bites, stress, and insomnia. These results are broadly in line with previous ethnobotanical studies. *Cymbopogon citratus* essential oils, aerial parts, and leaf extracts have been traditionally used against dysmenorrhea, fever, cough, and anxiety [56,57]. Of note, the genus *Cymbopogon* and specifically *Cymbopogon citratus* have a wide variety of biologically active phytochemicals and these bioactives have been examined for their antioxidant, antibacterial, and antidiabetic properties both in vivo and in vitro [58,59,60]. *Phyllanthus niruri* L. is the second most often utilized plant in the current study, and the participants who were surveyed strongly linked this plant with the management of diabetes. *Phyllanthus niruri* L. is a small herb that has been used by traditional medicine practitioners all over the world to treat kidney stones, gastrointestinal problems, liver affections, and diabetes [7,61,62]. Phytochemicals found in this species have been proven to possess antioxidant, hypoglycemic, and antihyperlipidemic properties [63,64]. The third most mentioned plant, *Momordica charantia* L., is also used for diabetes treatments by the population of Grande-Terre. The plant has many health-promoting qualities, and practically all its parts, including the fruits, have been traditionally used for diabetes management due to its anti-inflammatory, anti-diabetic, and anti-obesity effects [65]. Triterpenoids, steroidal saponins such as charantin, alkaloids such as vicine, peptides such us polypeptide-p, and flavonoids from *Momordica charantia* L can act as insulin sensitizers, insulin mimetics, α-glucosidase inhibitors, and glucose metabolism enzyme regulators [66,67]. A large proportion of the medicinal plants mentioned in our survey are also mentioned in ethnobotanical studies from all over the world. In Peru, *Passiflora edulis f. flavicarpa* is also used to treat hypertension [39]; in India, *Mirabilis jalapa* L. is also used to improve wound healing [48] and *Carica papaya* L. is used against inflammatory pain [35]; and in India and Spain, *Aloe vera* (L.) Burm.f. is used to cure skin problems or insect bites [45,46].

To select the relevant information, the data was filtered using different quantitative ethnobotanical parameters. ICF values ranged from 0 to 0.73, indicating that there is a low level of homogeneity and a certain lack of consensus in herbal remedy preparation in Grande-Terre. Values for Fa > 3% and Fi > 8% were established as filtering standards and only 22 plants of the 96 fulfilled the criteria. These results may indicate that numerous plants are utilized to treat the same ailment. Thus, these results show certain heterogeneity in the use of medicinal plants and the knowledge of the population in Grande-Terre. However, this diversity can also be attributed to a decrease in the quality and quantity of knowledge due to the loss of transmission between generations. According to the International Union for Conservation of Nature [20], only 3 species (*Capraria biflora* L., *Pimenta racemosa* (Mill.) J.W.Moore, and *Strumpfia maritima* Jacq.) are native taxa. Three other species have an uncertain biogeographical origin (*Lippia alba* (Mill.) N.E.Br. ex Britton & P.Wilson, *Justicia pectoralis* (Jacq.) J.F.Gmel. and *Peperomia pellucida* (L.) Kunth). The remaining 16 plants are introduced plant species. This means that 73 to 86% of the selected plants were brought from Africa, India, or Europe and gradually integrated into the cultural heritage of Guadeloupe. This characteristic underscores the fact that Guadeloupe is a crossroads where plants have been historically traded, together with the knowledge and cultural values attached to them [68]. Most of the knowledge came from family, so it can be inferred that the know-how in Guadeloupe is transmitted from generation to generation. Men and women were interviewed indistinctly and, interestingly, the transmitters mentioned were typically women, highlighting that women are the main owners of the knowledge and have a vital role in the information transfer in Grande-Terre.

Regarding the parts of the plants used, the aerial portions of the plants, specifically the leaves, were the most frequently cited. One possible explanation for the use of leaves is that leaves are typically the most edible portion of the plant. According to earlier research findings from comparable ethnobotanical investigations carried out in different countries throughout the world, the aerial parts and leaves appear to be the most used plant parts to prepare herbal treatments [10,69,70,71]. Nevertheless, compelling literature highlights the relevance of fruits and roots in herbal medicine [72,73]. The two principal modes of preparation of herbal remedies are infusion and decoction, and remedies are mainly taken orally. These results are generally consistent with related ethnobotanical studies, where infusions and decoctions are the most typical methods of making plant-based preparations [74,75,76].

Due to insularity and its geopolitical situation, the Guadeloupean population largely depends on imported medicines and our study provides supportive evidence that folk medicine is highly relevant in the Guadeloupe Archipelago. This study spotlights alternatives to pharmaceutical drugs and helps the local population to be less dependent on the import of medicine. The present study will thus contribute to the valorization and preservation of Guadeloupe’s traditional and natural heritage.

## 4. Materials and Methods

### 4.1. Survey Area

The survey was carried out in Guadeloupe, a French overseas region. The Guadeloupean archipelago is located at 16°15 N latitude and 61°35 W longitude, at a distance of 6732 km from Paris, 180 km from Martinique, and 2500 km from Florida, and hosted 394.110 inhabitants in 2016. With an area of 1628 km^2^ and less than 0.3% of the national territory, Guadeloupe is the second smallest French region after Martinique, five times smaller than the smallest region in mainland France, according to the Environment, Land Planning and Housing Department.

### 4.2. Ethnobotanical Survey

The present survey was conducted in French and Creole, the official and local languages, respectively. Interviews were performed during two different periods: June 2019 and March 2020. Face-to-face interviews were performed in 118 families randomly selected in 9 different municipalities of Grande-Terre Island: Le Gosier, Saint François, Sainte Anne, Morne à L’eau, Le Moule, Port-Louis, Anse Bertrand, Petit Canal, and Les Abymes (Figure 1). These communities consist of rural areas, urban areas, or both depending on each community. To obtain an overview of the use of medicinal plants by the local population, participants were chosen randomly without any regard for origin, gender, age, or previous knowledge in the field.

The ethnobotanical survey methodology was inspired by the TRAMIL protocol [23]. The interviews consisted of three phases: (I) Prior informed consent was obtained, (II) the interviewers asked the participants about ailments, one by one, (III) participants’ details were collected (age, place of birth, time lived in Guadeloupe, urban or countryside lifestyle, the origin of their knowledge, the origin of the plants, etc.). The list of ailments was elaborated by a local multidisciplinary group including doctors, pharmacists, botanists, statisticians, chemists, biologists, and social workers.

To avoid bias in the ethnobotanical survey responses, the survey was made of open questions and revolved around ailments rather than plants. In this context, the first interview question was: “What did you first do the last time this health problem happened in the family?” If the first treatment was an herbal remedy, the description of the plant was requested, including all the details concerning its uses, the part of the plant used, the way the remedy was prepared, the dose used, and if there were any related contraindications. This procedure was repeated for each selected ailment. If the participant used several plants to cure the same ailment, the questions were repeated for each plant. To attenuate the gender-related bias due to the embarrassment to speak about a gender-related disorder in front of the other gender (e.g., postnatal disorder in front of men or erectile dysfunction in front of women), the interview team was made of mixed pairs (one male and one female). Preferably, the pair was composed of a non-local and a local interviewer to lower mistrust toward the foreigner or the local. Finally, each interview locale was located geographically by GPS in case the interviewer team had to return to collect more information.

In many cases, plants were shown by the participants fresh from their garden or dry in dry preparations. Most of the plants named during the interviews were already known by the interviewers and a picture was taken of each botanical species when cited for the first time. Links between Creole and botanical names were validated thanks to the illustrated flora of phanerogams of Guadeloupe and Martinique [77] and the Caribbean herbal Pharmacopeia [34], the website World Flora Online (http://www.worldfloraonline.org/, accessed on 14 November 2022) in collaboration with TRAMIL Network (www.T and the herbarium of INRAE (Guadeloupe). Wherever there was doubt about a botanical name, the Creole name and the information given by the participant together with pictures of the plants were submitted to Dr. Alain Rousteau, a botanist at the University des Antilles. If the identification was uncertain, the plant was declared indeterminate.

### 4.3. Quantitative Ethnobotanics

The most widely used ethnobotanical parameters were utilized to analyze the data collected in the ethnobotanical survey.

#### 4.3.1. Minimum Sample Size

The number of persons to be surveyed, (n), was defined based on the number of families in the investigated region [28,78]. The equation for calculating the minimum number of participants is the following:

$$n=\frac{N\times Z_{\alpha \;}^{2}\times p\;}{d^{2}\left(N-1\right)}+Z_{\alpha \;}^{2}\times p\times q$$

where n is the minimum sample size, N is the total population size, Z is the fractile of the normal distribution corresponding to α with 90% confidence (α = 0.10; Z(α)^2^ = 1.64), p is the expected proportion of the country population (10%), q = 1, p = 1 − 0.1 = 0.9, and d is the precision (5%).

#### 4.3.2. Informant Consensus Factor (ICF)

The informant consensus factor (ICF) is an index used to measure the homogeneity of the participants’ knowledge [79]. When the ICF value is near 0, it means the participants disagree on the use of a plant to cure a specific ailment or that plants are picked randomly. In contrast, when the ICF value is near 1, this indicates that the plant is widely used by the participants. The ICF value is calculated using the following formula:

$$\mathrm{ICF}=\frac{\mathrm{Nur}-\mathrm{Nt}}{\mathrm{Nur}-1}$$

where Nur is the number of citations of use in each category and Nt is the number of different species used in this category. The minimal ICF chosen to consider certain homogeneity in the knowledge for each ailment was 0.55.

#### 4.3.3. Species Use Value (UV)

The relative importance of a plant species used as medicine in the study area was calculated with the help of the use value (UV) [29,30,79]. The UVs indicate the relative importance of each species known locally, and give information about the species that are considered most important in traditional ethnobotanical knowledge. The UVs are defined by the number of uses and the number of participants interviewed for a given species, according to the following equation:

$$UV=\frac{\Sigma U}{N}\times 100$$

where ΣU is the number of reported uses cited by each participant for a given plant species and N is the total number of participants interviewed.

#### 4.3.4. Frequency Use of a Plant for an Ailment (Fi)

The frequency Fi determines the plant species that is most frequently used to treat a particular ailment [32]. The plant species with the highest Fi value is considered the plant most frequently used for a given ailment. It is calculated using the following formula:

$$Fi=\frac{Ni}{Np}\times 100$$

where Fi is the frequency (%), Ni is the number of use-reports cited for a given plant species, i for a particular ailment, and Np is the total number of use-reports cited for any given species to treat this disease. To avoid anecdotal uses, a minimal frequency of 8% was chosen.

#### 4.3.5. Frequency of Treatment with Plants for Ailments (Fa)

For a given affliction, the frequency Fa indicates if an ailment is commonly cured with plants or if the treatment by the plant is anecdotal [32]. It is calculated with the following formula:

$$Fa=\frac{Na}{Nr}\times 100$$

where Fa is the frequency (%), Na is the number of remedies mentioned for a given affliction, and Nr is the total number of remedies given by the participants for all afflictions.

To eliminate the afflictions which are not commonly cured by plants, we decided to select only the ailments cured with plants by more than 1 in 8 participants, so by more than 12.5% of the participants. This minimum threshold leads to a minimal Fa of 3%.

## 5. Conclusions

The current research represents the first written recording of popular knowledge about medicinal plants in Grande-Terre, highlighting the relevance of popular knowledge of medicinal plant uses.

This study sheds light on the ailments that are treated using herbal remedies by the population of Grande-Terre. After data filtering and processing, 22 species belonging to 20 families and only 182 herbal remedies were identified. Among all the conditions mentioned, only cold/flu, hit/sprain, stomach ache/gas/bloating/digestion, diarrhea, insect bites, diabetes, stress/insomnia and hypertension are commonly managed with plant-based treatments. Lamiaceae, Poaceae, Myrtaceae, Cucurbitaceae and Rubiaceae appear as the most cited plant families—and *Cymbopogon citratus* (DC.) Stapf, *Phyllanthus niruri* L., and *Momordica charantia* L. as the most cited plant species. Furthermore, this study details the most used plant species for each affliction, providing useful information on the folklore knowledge for each specific ailment. The leaves are the plant portion that is utilized the most frequently across all herbal treatments, and infusion is the most popular mode of herbal remedy administration.

## Figures and Tables

**Table 1:** Ailments and plants identified in the ethnopharmacological survey. Selected ailments are shown with their respective ICF and Fa, and the plants most used to treat them with their respective Fi. * Germosén-Robineau, L. Pharmacopée Végétale Caribéenne. 3ème. TRAMIL. 2014.

Selected Ailment	ICF	F_a_	Selected Plant (Fi > 9%) (Voucher *)	Amount of Citations for This Ailment	Fi
Hypertension	0.59	7.4%	*Passiflora edulis* *(Vernon, H0933, GUAD)*	5	13.2%
			*Spondia cytherea* *(Fournet, H212, GUAD)*	5	13.2%
			*Peperomia pellucida* *(Longuefosse & Nossin, 27, HAVPMC)*	*4*	10.5%
			*Terminalia catappa* *(Girón, 229, CFEH)*	4	10.5%
Diabetes	0.59	9.4%	*Moringa oleifera (Fournet, H3153, GUAD)*	9	18.8%
			*Tinospora crispa* *(Lurel, H6328, GUAD)*	8	16.7%
			*Phyllanthus niruri (Moinard, H4891, GUAD)*	6	12.5%
			*Momordica charantia* *(Pimentel, 1111, JBSD)*	6	12.0%
Insect bites	0.60	3.1%	*Aloe vera* *(Pimentel, 1111, JBSD)*	4	25.0%
			*Citrus aurantiifolia* *(Rouzier, 172, SOE)*	4	25.0%
			*Cymbopogon citratus* *(Faujour, 9, BAR)*	3	18.8%
			*Pimenta racemosa* *(Jiménez, 60, JBSD)*	2	12.5%
Hit, muscular aches, sprain	0.64	5.7%	*Mirabilis jalapa* *(Zanoni, 42676, JBSD)*	9	65.5%
			*Carica papaya* *(Girón, 227, CFEH)*	7	24.1%
			*Justicia pectoralis* *(Fuentes, 4758, ROIG)*	4	13.8%
Stress, insomnia	0.71	3.5%	*Annona muricata (COVA 002, UA)*	11	61.1%
			*Lippia alba* *(Gimenez, 34, VEN)*	2	11.1%
			*Cymbopogon citratus* *(Faujour, 9, BAR)*	11	11.1%
Cold, flu	0.72	18.5%	*Cymbopogon citratus* *(Faujour, 9, BAR)*	19	20.0%
			*Plectranthus amboinicus (Fournet, H789)*	11	11.6%
			*Alpinia zerhumbet* *(Longuefosse&Nossin, 1, HAVPM)*	8	8.4%
Diarrhea	0.73	5.3%	*Psidium guajava* *(Delens, 23, VEN)*	10	59.3%
Stomach ache, gas, digestion, bloating	0.73	20.9%	*Strumpfia marítima (Fournet, H3392, GUAD)*	12	11.2%
			*Capraria biflora* *(Fournet, 4213, GUAD)*	9	9.3%
			*Momordica charantia* *(Pimentel, 1111, JBSD)*	16	8.4%

**Table 2:** Plant-based remedies in Grande-Terre. Le: leaves; Fl: flower; Fr: fruit; Bu: bud; Ro: root; Se: seed; Ae: aerial part; CI: contraindication (mainly for children and pregnant women); PW: pregnant woman; Ch: children; N.S: non stated. * Germosén-Robineau, L. Pharmacopée Végétale Caribéenne. 3ème. TRAMIL. 2014.

Scientific Name (Family, Voucher *)	Vernacular Names	Parts Used	CI	UV_s_	Fi	Ailment	Preparations Mode of Administration	Similar Use References
*Cympobogon citratus* (Poaceae) *(Faujour, 9, BAR)*	Sitwonnèl, ti can, Citronelle	Le	PW	25.40%	20%	Cold, flu	Leaves are the part used, mainly prepared in infusion. One cup has to be taken at night before going to bed.	[8,34,35]
					25%	Insect bites	Leaves, used in fumigation to ward off insects, have a preventive role.	
					11%	Stress, insomnia	Two or three leaves are used to prepare one cup of infusion, to be taken at night before going to sleep.	
*Pimenta racemosa* (Myrtaceae) *(Jiménez, 60, JBSD)*	Bwadenn Boisd’inde	Le; Fr	PW and Ch (alcohol)	2.50%	12.50%	Insect bites	Leaves or berries are macerated in rum for several days and the preparation is applied directly on the bite.	[35,36]
*Moringa oleifera* (Moringaceae) *(Fournet, H3153, GUAD)*	Moringa	Le; Fl; Fr; Se	N.S	14.40%	18.80%	Diabetes	One seed is eaten in the morning every day or one handful of leaves is used to make a cup of infusion, drunk every morning fasting. The leaves are sometimes also cooked and eaten as vegetables.	[34,35,36,37,38]
*Psidium guajava* (Myrtaceae) *(Delens, 23, VEN)*	Goyav, Gwayav	Le; Fr; Bu	N.S	13.60%	59.30%	Diarrhea	Two or three young leaves of buds are necessary to make one cup of infusion. The dose depends of the symptom but no more than two or three cups by day should be taken to avoid constipation risks.	[34,35,36,39]
*Momordica charantia* (Cucurbitaceae) *(Pimentel, 1111, JBSD)*	Pawoka	Le; Fr	PW	16.90%	8.40%	Clearing the liver and digestive system, digestion disorder	Leaves are used to prepare infusion.It is taken time to time to clear the liver and digestive system or one cup taken once or twice a day in case of digestion disorder, liver problems and hangover.	[34,35,36,40,41,42,43,44]
					12.50%	Diabetes	A handful of leaves or one fruit is used to prepare one cup of infusion, to drink once a day. Leaves are sometimes also cooked as vegetable and eaten with rice.	
*Aloe vera* *(Aloeaceae)* *(Jiménez, 1525, JBSD)*	Aloé	Le	N.S	16.1%	25%	Insect bites	Gel contained in its leaves is directlyapplied on the bites to relieve itch or pain.	[34,35,36,45,46,47]
*Alpinia Zerhumbet* (Zingiberaceae) *(Longuefosse & Nossin, 1, HAVPM)*	Atoumo	Le; Fl	N.S	8.50%	8.40%	Flu	Leaves, flowers or aerial parts are prepared in infusion and a cup has to be taken two or three times a day.	[34]
*Plectranthus amboinicus* (Lamiaceae) *(Medina, 47, CICY)*	Gwo ten	Le	Dosage Ch	13.60%	11.60%	Cold, flu, cough	Two or three leaves are used to make one cup of infusion. It has to be taken once or twice a day, at night or during the day, for 1 or 2 days.	[34]
*Mirabilis jalapa* (Nyctaginaceae) *(Zanoni, 42676, JBSD)*	Belle de nuit, bel dinuy	Ae	PW if used with alcohol	7.60%	65.50%	Sprain	Leaves are mashed, mixed with salt, sometimes rum or vinegar. The mixture is directly applied on the skin, maintained with a bandage and changed every day until healed.	[34,35,48]
*Justicia pectoralis* (Acanthaceae) *(Fuentes, 4758, ROIG)*	Zèbchapantyé	Le	PW and Ch (alcohol) N.S Le used alone	5.90%	13.80%	Sprain	Leaves are mashed, mixed with salt and sometimes castor oil. The mixture is directly applied on the skin, maintained with a bandage and changed every day until healed.	
*Caria papaya* (Caricaceae) *(Girón, 227, CFEH)*	Pyépapay	male Fl	PW (alcohol)	9.30%	24.10%	Hit, muscular aches	The male flowers are macerated for several days with bay rum, a distillate made with rum and leaves or berries of *Pimenta racemosa*. The preparation goes directly on the skin, and massage and friction applied on the painful area.	[34,35,39]
*Strumpfia maritima* (Rubiaceae)	Womarenbòlanmè	Le	PW and Ch (alcohol) Dosage Ch (infusion)	11%	11.20%	Stomach ache, intoxication	An infusion is prepared with the leaves of this indigenous plant and one cup has to be taken after the meal or at night. Leaves can also be marinated in rum—and if in pain, a spoon of the preparation is taken.	
*Capraria biflora* (Scrophulariaceae) *(Fournet, 4213, GUAD)*	Tépéyi	Le	Dosage Ch	11.90%	9.30%	Liver disorder, digestion problems, stomach ache	Three leaves or a small stem including aerial part are used to prepare one cup of infusion. The infusion can be taken in the morning, at night or several times during the day.	[34]
*Citrus aurantiifolia* (Rutaceae) *(Rouzier, 172, SOE)*	Citron sitwonpéyi	Le; Fr	N.S	11.90%	25%	Insect bites	Juice is applied on the bite.	[34,35]
*Tinospora crispa* (Menispermaceae)	Lyannsépan	Le	N.S	7.60%	16.70%	Diabetes	A piece of stem is infused in cold water and the infusion is taken either only in the morning or all day long during several days as a cure.	[49,50]
*Phyllanthus niruri* (Phyllantaceae) *(Moinard, H4891, GUAD)*	Grennanba fey	Ae	PW	21.20%	12.50%	Diabetes	Two or three leaves are used to make one cup of infusion to drink from time to time, or no more than two mornings in a row in emergency.	[38,39,51]
*Lippia alba* (Vervenaceae) (Fournet, H5210, GUAD)	Brizé Twa tas	Ae	N.S	9.30%	11.10%	Stress, insomnia	Two or three leaves are used to prepare one cup of infusion, to be taken at night before going to sleep.	[34,39]
*Annona muricata* (Annonaceae) *(COVA 002, UA)*	Kowosòl	Le	N.S	13.60%	61.10%	Insomnia	Two or three leaves are used to prepare one cup of infusion, to be taken at night before going to sleep.	[39]
*Passiflora edulis* (Passifloraceae) *(Vernon, H0933, GUAD)*	Marakoudja	Fr	N.S	4.20%	13.20%	Hypertension	Fruit is eaten raw or consumed in juice, once a day. Another way to use it is to macerate half of a fruit in warm water and drink one cup a day for three days.	[39]
*Peperomia pellucida* (Piperaceae) (Fournet, H1039, GUAD)	Koklaya	Ae	N.S	9.30%	10.50%	Hypertension	Leaves are consumed raw in salad or prepared in infusion (two or three leaves per cup) taken throughout the day.	[34]
*Spondias Cytherea* (Anacardiaceae) *(Fournet, H212, GUAD)*	Pòmnsitè	Fr	N.S	4.20%	13.20%	Hypertension	Fruits are consumed in juice from time to time during the day.	
*Terminalia catappa* (Combretaceae) (*Girón, 229, CFEH*)	Pyézanmann	Le	N.S	4.20%	10.50%	Hypertension	One red or yellow leaf is put in to the patient’s socksunder the arch of their feet, or on their head under their cap all day long. Leaves are also used in decoctions: half a leaf is used to prepare one cup, to be drunk once a day.	[35,37,52]

## Data Availability

Not applicable.
